# Prevalence of Nutritional Anaemia in Type 2 Diabetes Mellitus in the Absence of Renal Impairment

**DOI:** 10.7759/cureus.72946

**Published:** 2024-11-03

**Authors:** Suresh Kanna Subramaniam, Umashankar R., Vinatha M. C., Aravind Raj, Keerthana I., V. Ramachandra Rao

**Affiliations:** 1 Department of General Medicine, Sree Balaji Medical College and Hospital, Chennai, IND; 2 Department of Internal Medicine, Sree Balaji Medical College and Hospital, Chennai, IND

**Keywords:** anemia, balaji medical college study, diabetes mellitus, glycemic control, hematology investigations

## Abstract

Background: Anemia is more common in people with type 2 diabetes mellitus (T2DM) and is more likely when glycemic control is poor. Anemia in T2DM can reduce quality of life and increase cardiovascular risk, and therefore, its prevalence and contributory factors in diabetic patients with normal kidney function are important to understand.

Aim and objectives: The aim of this study is to assess the prevalence of anemia in individuals with type 2 diabetes and normal kidney function, focusing on blood glucose, serum creatinine, HbA1c, hemoglobin, and hematocrit levels. The research also examined the influence of gender, age, and glycemic control on anemia occurrence.

Materials and methods: This study was conducted at Balaji Medical College and General Hospital from March 2021 to January 2023 to evaluate blood glucose, HbA1c, serum creatinine, hemoglobin, and hematocrit levels in type 2 diabetes patients. Data were collected using a standardized proforma, including age, sex, occupation, physical activity, and clinical history. Biochemical and hematology analyses were performed on venous blood samples collected via venipuncture. Statistical analysis used an unpaired two-tailed t-test with a significance level of p < 0.05. The study included 100 patients categorized by glycemic control, gender, age, and socioeconomic status.

Results: Results showed a high prevalence of anemia in uncontrolled diabetic patients (mean hemoglobin level 11.75 ± 1.72 g/dL vs. 14.46 ± 1.445 g/dL in the controlled cases, p = 0.0001). The mean hematocrit level was 35.46 ± 5.136% in uncontrolled patients compared to 43.62 ± 4.59% in controlled patients (p = 0.0001), showing a direct relationship between poor glycemic control and low hematological indices.

Conclusion: The study shows that anemia is common in people with type 2 diabetes, especially if their blood sugar is not well controlled or as they get older. It highlights the need for regular blood tests to check for anemia in diabetes patients, especially older ones. The study recommends adding iron supplements, balanced diets, and vitamins to treatment plans. These steps can help manage both diabetes and anemia better, leading to improved patient care and outcomes.

## Introduction

Type 2 diabetes mellitus is characterized by hyperglycemia, insulin resistance, and insufficient insulin production, marking it as a chronic metabolic disorder. The prevalence of diabetes has witnessed a concerning surge globally, transforming it from a condition primarily affecting the elderly into a leading cause of morbidity and mortality among individuals in their 20s and 30s over the past three decades. This escalation is not confined to specific regions but appears to be a worldwide phenomenon. Although both type 1 and type 2 diabetes diagnoses have increased, it is the latter that predominantly drives the pandemic, constituting over 90% of all diabetes cases [1]. India has witnessed a significant rise in diabetes cases, earning the country the dubious distinction of having the highest prevalence of the disease globally.

In 2000, the World Health Organization revealed that about 32 million people in India had diabetes [2]. A subsequent report from IDF (2015) predicted that 40.9% of the population in India was affected by diabetes, and projections show a scenario where, by 2025, this figure will have risen to 69.9%. The explosion of diabetes cases in India mirrors the global trend, thus indicating the need for immediate implementation of management techniques. Complications emanating from type 2 diabetes are various, but renal failure plays a prominent part. Symptoms such as increased thirst, frequent urination, unexplained weight loss, increased appetite, fatigue, and delayed wound healing indicate hyperglycemia, insulin resistance, and insulin deficiency, characteristic features of type 2 diabetes [3]. Long-term consequences may include cardiovascular disease, stroke, diabetic retinopathy-induced blindness, and peripheral vascular disease, necessitating amputations if blood sugar levels remain high [4]. Furthermore, hyperglycemia can cause acute ketoacidosis and hyperosmolar coma, underscoring the importance of controlling glucose levels when managing diabetes. Insulin, produced by beta cells in the pancreas, facilitates glucose uptake by cells for energy production. However, in type 2 diabetes, cells exhibit reduced responsiveness to insulin, impeding glucose absorption and resulting in elevated blood sugar levels, termed hyperglycemia.

It is important to note that type 2 diabetes does not only occur in overweight individuals but also among lean and old people. Risk factors for developing type 2 diabetes include genetic predisposition, sedentary lifestyle, poor eating habits, and abdominal obesity. This makes type 2 diabetes, rather than hypertension, the primary cause of end-stage renal disease (ESRD), as it is linked to a higher incidence of renal complications. Anemia and diabetic nephropathy are often concomitant conditions that exacerbate both microvascular and macrovascular complications. It should be noted that borderline low hemoglobin may indicate relatively more susceptibility to microvascular problems, morbidity, and mortality in patients with diabetes [5]. The connection between anemia and diabetic nephropathy highlights the intricate relationship between renal impairment as well as various other complications due to diabetes. The effects of anemia go beyond physiological manifestations, affecting quality of life and increasing morbidity [6,7]. Fatigue, dyspnea, dizziness, anorexia, cognitive impairment, and reduced exercise tolerance are some of the debilitating symptoms accompanying anemia with concurrent renal dysfunction among patients with diabetes mellitus. The escalating prevalence of type 2 diabetes underscores the urgent need for comprehensive management strategies to mitigate its complications, including renal failure and anemia. Effective glycemic control, lifestyle modifications, early screening, and timely intervention are imperative to alleviate the burden of diabetes and improve patient outcomes. Apart from biological and lifestyle risk factors, socioeconomic status has a substantial role in the prevalence of nutrient deficiencies and anemia. The fact that individuals who belong to a lower socioeconomic background tend to be more prone to anemia derives from a lack of access to nutrient-rich foods, health education, and health care.

The objective of this study is to measure and analyze key clinical markers, including blood glucose, serum creatinine, glycated hemoglobin (HbA1c), hemoglobin, and hematocrit, among individuals with type 2 diabetes and normal kidney function. This study aims to investigate the prevalence and probability of anemia in patients with type 2 diabetes to see if gender and age affect anemia in this population. The research also seeks to ascertain the role that glycemic management plays in the prevalence of anemia and how this can be used in the advancement of treatment protocols for the management of anemia in diabetic patients. This study hopes to better inform more effective therapeutic approaches for individuals with diabetes and anemia through understanding the interplay between glycemic control and anemia. This study establishes a baseline for further investigations, including studies with larger sample sizes.

## Materials and methods

Study design and location

This study took place at Balaji Medical College and General Hospital, spanning from March 2021 to January 2023. The goal was to evaluate key health indicators in patients with type 2 diabetes who visited the general medicine department. The study was approved by the Institutional Ethics Committee (Ref. No. 002/SBMCH/IHEC/2021/1543).

Inclusion criteria

The inclusion criteria for this study included adults aged 18 years or older, diagnosed with type 2 diabetes mellitus, normal kidney function verified by serum creatinine levels within standard ranges, and informed consent provided by all participants.

Exclusion criteria

The exclusion criteria for this study included individuals with unstable cardiovascular or circulatory conditions, chronic illnesses other than diabetes (e.g., cancer, chronic liver disease), recent history of significant blood loss, any form of anemia not associated with diabetes, kidney failure, or impaired renal function, use of medications that could affect glucose or hemoglobin levels, and pregnancy.

Data collection

The study included 100 patients with type 2 diabetes, grouped by blood sugar control, gender, and age. All the relevant information, such as age, gender, occupation, physical activity level, socioeconomic status, and medical history, was documented on a standardized form. This ensured that all important details were recorded accurately.

Sample collection and handling

A total of 100 patients were divided into two groups: controlled diabetic cases (n=37) and uncontrolled diabetic cases (n=63). Blood samples were taken from the patients using disposable syringes. About 5 mL of blood was drawn from the vein in the arm and placed in clean test tubes. The blood was left to clot for 10-15 minutes before being spun in a centrifuge at 3000 rpm for four to five minutes to separate the serum for further tests.

Biochemical and hematological analysis

We measured glucose levels in the blood serum to evaluate blood sugar control, which is crucial for assessing diabetes management. HbA1c was tested to provide an average of blood glucose over the past few months, indicating long-term glycemic control. Hemoglobin and hematocrit levels were analyzed to check for anemia, as they reflect red blood cell (RBC) count and oxygen-carrying capacity. Serum creatinine levels were measured to assess kidney function, as elevated levels can signal kidney impairment, which may worsen anemia by affecting erythropoietin production. All serum samples were stored at −20 °C for later analysis to keep them intact.

Statistical analysis

The collected data were analyzed using GraphPad software (GraphPad Software, Inc., La Jolla, CA) with an unpaired two-tailed t-test to compare health indicators between patients with well-controlled and poorly controlled type 2 diabetes. Prior to the t-test, data normality was assessed using the Shapiro-Wilk test, and transformations were applied where necessary. A 95% confidence interval and a significance level of p < 0.05 were used to determine statistical significance, with Bonferroni correction applied to account for multiple comparisons. The analysis covered key health indicators, including blood glucose, HbA1c, hemoglobin, hematocrit, and serum creatinine levels, providing clear insights into the impact of diabetes control on these variables.

The Shapiro-Wilk test evaluates whether data is normally distributed. Its test statistic $W$ is calculated as:

$W = \frac{\left( \sum_{i=1}^{n} a_i x_{(i)} \right)^2}{\sum_{i=1}^{n} \left( x_i - \bar{x} \right)^2}$

where $x_{(i)}$ are the ordered sample values; $\bar{x}$ is the sample mean; ​​​​​$a_i$ are constants derived from the covariance matrix of the ordered sample values; ${n}$ is the sample size.

## Results

Age distribution

The age distribution of the subjects is presented in Table 1. The mean age for the controlled type 2 diabetes mellitus (T2DM) group was 55 ± 6.531 years, and for the uncontrolled T2DM group, it was 54.84 ± 7.197 years. The difference in age distribution between the two groups was not statistically significant (p=0.91).

**Table 1:** Distribution of age in different groups significant value (p) < 0.05

Groups	Age	Mean ± SD	P-value	T-value
Controlled T2DM	55	55 ± 6.531	0.91	−0.13
Uncontrolled T2DM	54.84	54.84 ± 7.197	-	-

Fasting blood glucose levels

**Figure 1:**
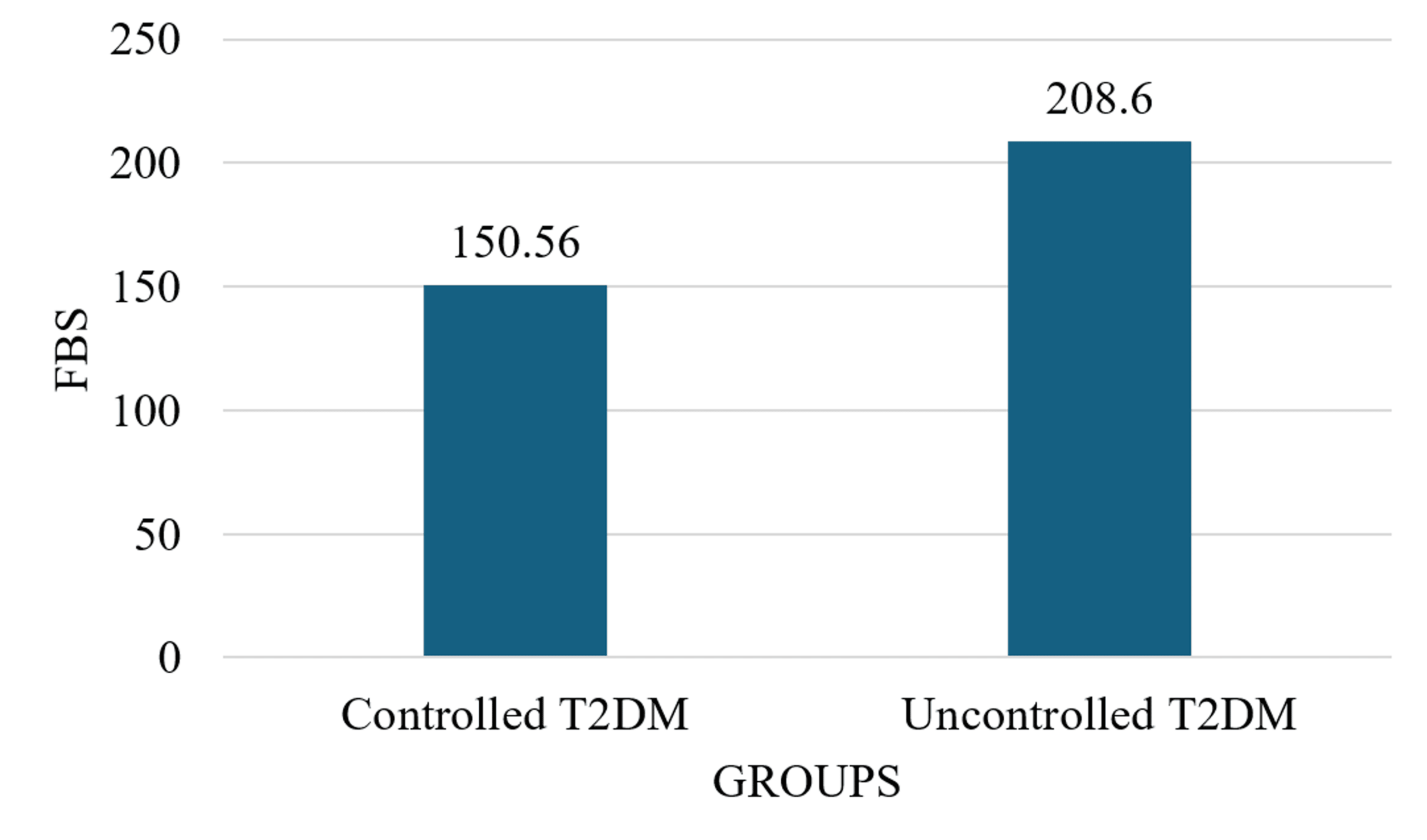
Graphical representation of FBS level distribution in different groups

Fasting blood glucose levels showed a significant difference between the two groups, as detailed in Table 2. The controlled T2DM group had a mean fasting blood glucose level of 150 ± 22.40 mg/dL, while the uncontrolled T2DM group had a mean of 208.6 ± 51 mg/dL. This difference was statistically significant (p=0.0001) (Figure 1).

**Table 2:** Distribution of FBS levels in different groups Significant value (p) < 0.05

Groups	FBS	Mean ± SD	P-value	T-value
Controlled T2DM	150.56	150 ± 22.40	0.0001 (7.837)	7.837
Uncontrolled T2DM	208.6	208.6 ± 51	-	-

HbA1c and hemoglobin levels

**Figure 2:**
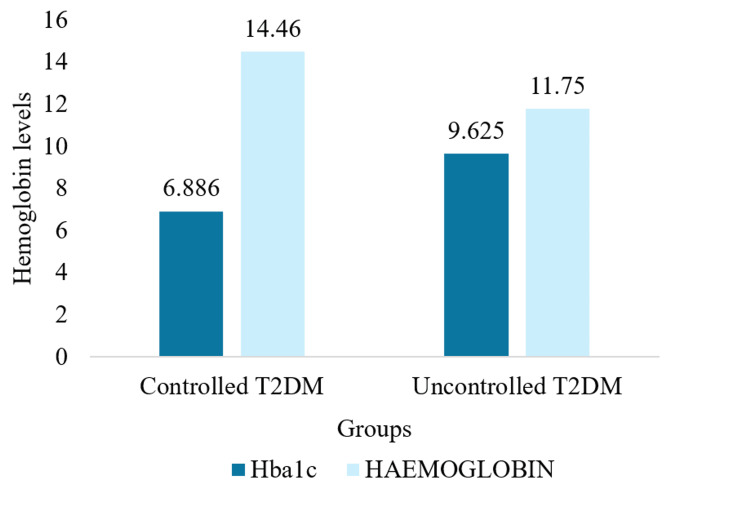
Graphical representation of of glycated haemoglobin levels and haemoglobin levels in controlled and uncontrolled T2DM groups

Table 3 presents the levels of glycated hemoglobin and hemoglobin in both groups. Glycated hemoglobin was significantly lower in the controlled T2DM group (6.88% ± 0.702%) compared to the uncontrolled T2DM group (9.625% ± 1.65%) with a p-value of 0.0001. Hemoglobin levels were also significantly higher in the controlled group (14.46 ± 1.445 g/dL) compared to the uncontrolled group (11.75 ± 1.72 g/dL), with a p-value of 0.0001 (Figure 2).

**Table 3:** Distribution of glycated hemoglobin levels and hemoglobin levels in different groups Significant value (p) < 0.05

Group	HbA1c	Mean ± SD	P-value	T-value
Controlled T2DM	6.886	6.88 ± 0.702	0.0001 (11.55)	11.55
Uncontrolled T2DM	9.625	9.625 ± 1.65	-	-
Group	Hemoglobin levels	Mean ± SD	P-value	T-value
Controlled T2DM	14.46	14.46 ± 1.445	0.0001 (8.066)	8.066
Uncontrolled T2DM	11.75	11.75 ± 1.72	-	-

Serum creatinine and hematocrit levels

Table 4 shows the distribution of serum creatinine and hematocrit levels. Serum creatinine levels were significantly lower in the controlled T2DM group (0.945 ± 0.177 mg/dL) compared to the uncontrolled T2DM group (1.134 ± 0.198 mg/dL) with a p-value of 0.0001. Hematocrit levels were also higher in the controlled T2DM group (43.62 ± 4.59%) compared to the uncontrolled T2DM group (35.46 ± 5.136%) with a p-value of 0.0001.

**Table 4:** Distribution of creatinine levels and hematocrit levels in different groups Significant value (p) < 0.05

Group	Average creatinine levels	Standard deviation	P-value	T-value
Controlled T2DM	0.945	0.9 ± 0.177	0.0001	4.663
Uncontrolled T2DM	1.134	1.134 ± 0198	-	-
Group	Average hematocrit levels	Standard deviation	P-value	T-value
Controlled T2DM	43.46	43.62 ± 4.59	0.0001	7.979
Uncontrolled T2DM	35.46	35.46 ± 5136	-	-

The study elucidated distinct differences in biochemical and hematological parameters between controlled and uncontrolled diabetes groups, underscoring the importance of glycemic control in managing diabetes-associated complications. These findings highlight the need for tailored therapeutic interventions aimed at optimizing glycemic control to mitigate the progression of diabetes and its associated complications. The following are the diagrammatic presentations of the various variables.

## Discussion

The study extensively discusses the prevalence of anemia among patients, particularly emphasizing the association between anemia and diabetes mellitus. Previously conducted studies found a remarkable frequency of anemia, particularly among patients with diabetes with renal impairment [8], which underscores the need for diagnosing anemia in diabetics. In addition, socioeconomic status is an important determinant of anemia prevalence. Those from lower socioeconomic backgrounds are at risk of anemia because of limited access to nutrient-rich foods, healthcare, and health education, which increases their risk factors for nutrient deficiencies and anemia. Anemia and diabetes are seen as major concerns for health based on their increased cardiovascular diseases, retinopathy, and stroke risks that affect the lifespan of patients [9-10]. The latter include the degree of albuminuria and renal impairment, while prevalence rates vary with gender in different studies. Notably, the mean hemoglobin level of uncontrolled diabetic patients was 11.75 ± 1.72 g/dL and was markedly lower than 14.46 ± 1.445 g/dL in controlled cases. Inappropriate glycemic control might play a role in this substantial difference and actually increase the risk for anemia-related complications.

Factors such as chronic diabetes, higher BMI, hypercholesterolemia, and poor glycemic control lead to a greater incidence of anemia, particularly among elderly individuals who may be limited by their diet and perhaps iron deficiency [11]. Gender differences pose a significant concern as more women than men suffer from anemia, primarily due to social factors such as lack of empowerment, low literacy levels, and poor diet, which can lead to malnutrition in certain societies. This finding is consistent with previous research that shows iron deficiency anemia is a common problem for women, owing in part to physiological reasons such as menstruation that increase the risk of iron depletion. Iron deficiency anemia may worsen the risk of health in type 2 diabetes, especially in the presence of poor glycemic control [12].

The focus of this study is on the importance of tailoring treatment guidelines for incorporating hematology assessments by age and glycemic control to optimize diabetes control in adult populations [13]. Anemia and diabetes mellitus are two terms that have been overused and extensively discussed in medical research. Patients with diabetes mellitus usually suffer from anemia, which is characterized by a low number of red blood cells or hemoglobin [14]. This condition complicates the management of diabetes as well as subjects affected persons to other health risks. There is a higher prevalence of anemia among diabetic people compared to the general population. The risk is increased owing to various factors, including the pathophysiology behind the disease itself [15]. Erythropoiesis, the process in which RBCs are formed in the bone marrow, may be altered due to CH, IR, and inflammation associated with DM. Apart from these factors, those suffering from diabetic nephropathy (a common renal complication affecting people with DM) exhibit reduced production of erythropoietin, a hormone required for red cell synthesis, thereby aggravating further the anemia [16].

Furthermore, certain comorbidities commonly seen in diabetic individuals, such as chronic kidney disease and peripheral vascular disease, can contribute to anemia through various mechanisms. Chronic kidney disease, for example, can impair the kidneys' ability to produce erythropoietin, leading to decreased red blood cell production [17]. Peripheral vascular disease may result in tissue hypoxia, stimulating erythropoiesis but also leading to increased red blood cell destruction. The subsequent persistent presence of anemia in diabetic patients not only worsens their overall health outcomes but also complicates the management of their diabetes [18]. Anemia can exacerbate symptoms of fatigue, weakness, and shortness of breath, which can further impair a patient's quality of life and ability to manage their diabetes effectively [19]. Moreover, anemia has been associated with an increased risk of cardiovascular events, mortality, and hospitalizations in diabetic individuals.

Limitations of the study 

Several limitations are associated with this study. Overall, using only 100 patients may not describe the range of variability within the diabetic population. This cross-sectional design also poses difficulties in making casual inferences about the relationships between the variables, and future longitudinal research has to be conducted to elucidate these associations more precisely. Further limitations included the inability to closely control other possible demographic, dietary, or medication interference with the anemia. Furthermore, questionnaire data is prone to recall bias in several aspects involving such self-reported demographic data. Further, larger samples from different centers and longitudinal studies are suggested to strengthen and generalize the findings.

## Conclusions

This research, in accordance with the objectives of the study, shows that anemia is common among type 2 diabetes patients, especially those with poor glycemic control and older age groups. These findings demonstrate the direct effect of glycemic control on the severity of anemia, with mean hemoglobin and hematocrit levels significantly lower in the uncontrolled diabetic group (11.75 ± 1.72 g/dL and 35.46 ± 5.136%, respectively) than in the controlled group. The study's implications stress the necessity of routine hematologic monitoring plus specific nutritional interventions, such as iron supplementation, for patients with diabetes, particularly those with low income. This research suggests that addressing anemia through integrated diabetes management protocols can enhance diabetic care outcomes, boost quality of life, and lower complication rates in patients with type 2 diabetes. These findings can be expanded upon in future studies looking at the longitudinal impacts of integrated anemia and diabetes management to refine therapeutic approaches better. In addition, this study demonstrates how socioeconomic factors are related to the prevalence of anemia in diabetic patients. Reducing anemia and improving patient outcomes among lower-income diabetic populations may require addressing socioeconomic barriers, such as access to nutritious foods and healthcare.
